# Identifying Breast Cancer Subtype Related miRNAs from Two Constructed miRNAs Interaction Networks in Silico Method

**DOI:** 10.1155/2013/798912

**Published:** 2013-11-20

**Authors:** Lin Hua, Lin Li, Ping Zhou

**Affiliations:** Biomedical Engineering Institute of Capital Medical University, Beijing 100069, China

## Abstract

*Background*. It has been known that microRNAs (miRNAs) regulate the expression of multiple proteins and therefore are likely to emerge as more effective targets of selective therapeutic modalities for breast cancer. Although recent lines of evidence have approved that miRNAs are associated with the most common molecular breast cancer subtypes, the studies to breast cancer subtypes have not been well characterized. *Objectives*. In this study, we propose a silico method to identify breast cancer subtype related miRNAs based on two constructed miRNAs interaction networks using miRNA-mRNA dual expression profiling data arising from the same samples. *Methods*. Firstly, we used a new mutual information estimation method to construct two miRNAs interaction networks based on miRNA-mRNA dual expression profiling data. Secondly, we compared and analyzed the topological properties of these two networks. Finally, miRNAs showing the outstanding topological properties in both of the two networks were identified. *Results*. Further functional analysis and literature evidence confirm that the identified potential breast cancer subtype related miRNAs are essential to unraveling their biological function. *Conclusions*. This study provides a new silico method to predict candidate miRNAs of breast cancer subtype from a system biology level and can help exploit for functional studies of important breast cancer subtype related miRNAs.

## 1. Introduction

Stratification of breast cancer patients according to their clinical subtype and prognosis is a desirable goal in breast cancer treatment in order to achieve a better personalized medicine. Although still in the early stages of research, molecular breast cancer subtypes may become useful in planning treatment and developing new therapies. As the most common subtype, luminal-A exhibited risk factors typically reported for breast cancer in previous studies, including inverse associations for increased parity and younger age at first full-term pregnancy [1]. As another important breast cancer subtype, basal-like exhibited several associations that were opposite to those observed for luminal-A, including increased risk for parity and younger age at first term full-term pregnancy [1]. In addition, some studies found women with multiple live births who did not breastfeed and women who used medications to suppress lactation were at increased risk of basal-like, but not luminal-A. From molecular biology level, it has been reported luminal-A and basal-like subtypes have distinct and reciprocal gene expression profiles as well as large differences in clinical characteristics, including survival [2]. Luminal-A is one of ER-positive subtype since it has an expression pattern similar to the luminal epithelial cells of the breast and luminal-A tumors tend to have the best prognosis [3]. In contrast, basal-like tumors are characterized by an expression signature similar to that of the basal/myoepithelial cells of the breast and are reported to be associated with aggressive behavior and poor prognosis [2, 4, 5]. Therefore, identification of breast cancer subtype related biomarkers is very important to help in finding new treatment strategies.

As novel biomarkers, miRNAs have been proven to be frequently deregulated in human breast cancer by recent studies [6, 7]. A large number of studies have suggested that miRNAs play essential roles in biological processes and might correlate with specific clinical features of breast cancer, such as estrogen and progesterone receptor expression, tumor stage, vascular invasion, and proliferation index. Therefore, the identification of miRNA expression-based breast cancer subtypes is considered a critical means of prognostication. With the rapid development of system biology methods, an increasing number of studies have prioritized some novel miRNAs related to breast cancer or breast cancer subtype as well as understanding their properties. By integrating different data type, such as microarray data, genotype data, DNA methylation data, and the network or pathway information, into the prognostic biomarker discovery, the prediction performance will be improved greatly. Indeed, the significant progress has been made for the identification and interpretation of the cancer-related miRNAs with the aid of system biology methods. For example, it has been reported that potential candidate disease-related miRNAs can be identified by comparing similarities between miRNAs with known molecular functions [8] or associated with specific disease [9]. Also, some studies inferred the functions of miRNAs by analyzing the properties of miRNA targets [10]. Considering that the targeting propensity of miRNA can be largely explained by the functional behavior of protein connectivity in the protein-protein interaction network, Sun et al. proposed a novel miRFunSim method to quantify the associations between miRNAs in the context of protein interaction network [11]. Specifically, for the identification of important biomarkers of primary breast cancer subtypes, a recent advantage is combining genomic DNA copy number arrays, DNA methylation, exome sequencing, messenger RNA arrays, microRNA sequencing, and reverse-phase protein arrays to find each of breast cancer subtypes showing significant molecular heterogeneity [12]. Furthermore, a lot of integrative methods that combine the target-prediction algorithms with both mRNA and miRNA expression data have become popular. For example, Luo et al. performed a systematic evaluation of functional miRNA-mRNA interactions associated with the invasiveness of breast cancer cells using a combination of integrated miRNA and mRNA expression profiling, bioinformatics prediction, and functional assays [13]. Lionetti et al. identified the miRNA expression patterns and miRNA-mRNA regulatory network in distinct molecular groups of multiple myeloma using miRNA-mRNA dual expression profile data [14]. Zhang et al. integrated the miRNA and gene expression profiles in a multiple nonnegative matrix factorization framework to identify the miRNA-gene regulatory comodules [15]. Most of these methods used linear correlation coefficients to measure the relationship between miRNAs and their targets.

However, sometimes the Pearson correlation coefficient cannot detect a significant correlation when two variables are not in linear dependence. Fortunately, statistical correlation measures based on mutual information are able to capture more features of the data than the linear Pearson correlation coefficient [16]. Therefore, different from other studies, the joint analysis of miRNA-mRNA dual expression profiling data arising from the same samples provided here is using a newly developed mutual information estimation method to construct two miRNA interaction networks based on the expression profiling data of miRNA and its targets (mRNA), respectively. A comparison of topological properties between these two networks allowed us to identify some key miRNAs which have been confirmed to be associated with breast cancer subtype by recent evidence. Further functional analysis and literature evidence confirm that the identified potential breast cancer subtype related miRNAs are essential to unraveling their biological function. This study provides a new silico method to predict candidate miRNAs of breast cancer subtype from a system biology level and can help explore the functional studies of clinically important breast cancer subtype related miRNAs.

## 2. Materials and Methods

### 2.1. Data Source

In this analysis, we selected mRNA expression profiling data including 24,817 mRNAs (GSE19783) reported by Enerly et al. [17] to implement our analysis, while 15 basal-like samples and 41 luminal-A samples were included. For the miRNA expression profiling data, the original microarrays covered 799 miRNAs arising from the Agilent Technologies. miRNA expression status was scored as present or absent for each gene in each sample by default settings. miRNAs in samples that were run in replicate were considered present if scored in one of the two arrays. Those miRNAs that were detected in less than 10% of the samples were excluded. This filtering resulted in 489 miRNAs considered to be expressed in this set of human breast tumors. In the present study, we directly selected this filtered miRNA expression profiling data (GSE19536) provided by Enerly et al. [17] to implement our analysis. We considered the most basic microarray analysis approach, SAM (significance analysis of microarrays) [18], as a filter to extract statistically significant differential expression of miRNAs that distinguish the reciprocal basal-like and luminal-A breast cancer subtypes. In this method, repeated permutations of the data are used to determine if the expression of any miRNA is significant related to the phenotype. To get more information, *P* < 0.05 and false discovery rates (FDR) <0.1 is often as a popular and less stringent filter criterion to select a larger set of differentially expressed genes [19]. We therefore also used this criterion to determine miRNAs with various differentially expressed. According to this criterion, 201 differentially expressed miRNAs are identified and will be used for further analysis. We defined a breast cancer subtype related miRNA that is luminal-A trend when it is significant (*P* < 0.001 and FDR < 0.05) and shows higher expression in luminal-A sample than in basal-like sample. On the contrary, a miRNA is basal-like trend when it is significant (*P* < 0.001 and FDR < 0.05) and shows higher expression in basal-like sample than in luminal-A sample.

### 2.2. Construction of miRNAs Interaction Networks

#### 2.2.1. Construction of miRNAs Interaction Network Using miRNA Expression Profiling

In the practice, inferring large networks using mutual information (MI) has been shown to be an effective strategy. In this analysis, we used a newly developed mutual information estimation method, parmigene (parallel mutual information estimation for gene network reconstruction) [20], to construct miRNAs interaction network. This method implements a mutual information estimator based on *k*-nearest neighbor distances that is minimally biased with respect to the other methods and uses a parallel computing paradigm to reconstruct biology regulatory networks. For each triple consisting of nodes *i*, *j*, and *k*, this algorithm considers each edge of the triple independently and removes the weakest link if MI(*i*; *j*) < MI(*j*; *k*) − *ε* and MI(*i*; *j*) < MI(*i*; *k*) − *ε* according to the threshold. In this analysis, we selected 0.05 as the threshold to remove the weakest edge of each triple of nodes. The program was implemented in parmigene package of R software (http://www.r-project.org/). After assembling all reserved miRNA-miRNA pairs, miRNAs interaction network based on miRNA expression profiling data is constructed.

#### 2.2.2. Construction of miRNAs Interaction Network Using the Reconstructed miRNA Expression Dataset

We know that miRNA can act by binding to the complementary sites on the 3′ untranslated region (UTR) of the target gene to induce cleavage with near perfect complementarity or to repress productive translation [21]. Therefore, exploring the relationships between the targets of miRNAs might reflect partly the potential relationships between miRNAs. Based on this assumption, we constructed another miRNAs interaction network using the reconstructed miRNAs expression dataset. This process can be described as follows. Firstly, for each identified differentially expressed miRNA, we got its target genes from MicroCosm Targets database (http://www.ebi.ac.uk/enright-srv/microcosm/htdocs/targets/v5/), in which the candidate miRNA-target relationships were mostly predicted by miRanda algorithm [22]. Secondly, we defined an activity score for each miRNA as the summary of the expression values of all mRNAs targeted by this miRNA. In this analysis, we used principal component analysis (PCA) method to get the summary of all targets of each miRNA. The PCA technique can effectively characterize the internal structure of high dimension dataset by preserving the variance in the data while transforming the data into low dimension space. Finally, we extracted the first principal component from PCA which was used as the activity score for the corresponding miRNA. After assembling the first principal component (activity scores) of all miRNAs, the reconstructed miRNAs expression dataset was generated. In this dataset, each miRNA was expressed by a linear combination of the expressions of all its targets for each sample. For this reconstructed miRNAs expression dataset, we still used the mutual information estimation method, parmigene, as described above to construct the miRNAs interaction network.

### 2.3. Identification of Breast Cancer Subtype Related miRNAs from Two Constructed miRNAs Interaction Networks

In this study, we analyzed and compared the topological properties between two constructed miRNAs interaction networks. Generally, hubs in cellular networks are central players involving in broadly biochemical and genetic events [23]; we therefore focused our attention on those hubs. We calculated some topological properties of these hubs, such as betweenness and closeness. While betweenness is a centrality measure of a vertex within a graph, nodes that have a high probability to occur on a randomly chosen shortest path between two randomly chosen nodes also have a high betweenness [24]. Closeness is the reciprocal of the sum of all the geodesic (shortest) distances from a given node to all other nodes [24]. In other words, a miRNA with higher betweenness and higher closeness means that it is on higher number of shortest paths between miRNAs, and this miRNA is important [25]. Indeed, some studies have approved that the topological properties of disease genes are very different from those of nondisease genes [26, 27] in gene-gene network. For example, disease genes tend to interact with more genes than nondisease genes. These studies indicate that the gene-gene network can provide candidate genes for some diseases. Similarly, we assumed the miRNAs showing the outstanding topological properties in miRNA-miRNA network might be the potential disease miRNAs. Therefore, we focused on those common hub miRNAs showing the outstanding topological properties shared by these two constructed miRNAs interaction networks as candidate miRNAs and confirmed their potential importance in breast cancer subtype.

### 2.4. Comparison of Subtype Classification Performance

To evaluate the ability of the candidate miRNAs extracted from two constructed miRNAs interaction networks for discriminating breast cancer subtype, we defined two miRNA groups: one is the miRNAs group with the common hub miRNAs shared by two constructed miRNAs interaction networks and the other is the miRNAs group with 201 differentially expressed miRNAs. We applied four classifiers: naïve Bayes [28], *k*-nearest neighbor (*k*NN) [29], support vector machine (SVM) [30], and random forests (RF) [31] to compare the subtype classification performance of these two miRNAs groups when they are taken as predictor variables to classify samples. We used 5-fold cross validation to assess the classification accuracy rate of these different machine-learning methods. We set *k* at three in *k*-nearest neighbor program and took radial basis function (RBF) as the kernel function in the support vector machine program. For random forests program, 5,000 trees were constructed. Original miRNA expression dataset and the reconstructed miRNA expression dataset arising from PCA were used to implement this process, respectively. 

### 2.5. A Global Test for Candidate miRNAs Group

To explore whether the identified candidate miRNAs group extracted from two constructed miRNAs interaction networks is associated with breast cancer subtype, we used Goeman's global test here [32] to determine its significance. Global test can determine whether the global expression pattern of a group of genes (instead of miRNAs in our study) is significantly related to the clinical outcome.

### 2.6. Survival Analysis for Candidate miRNAs

**Figure 1:**
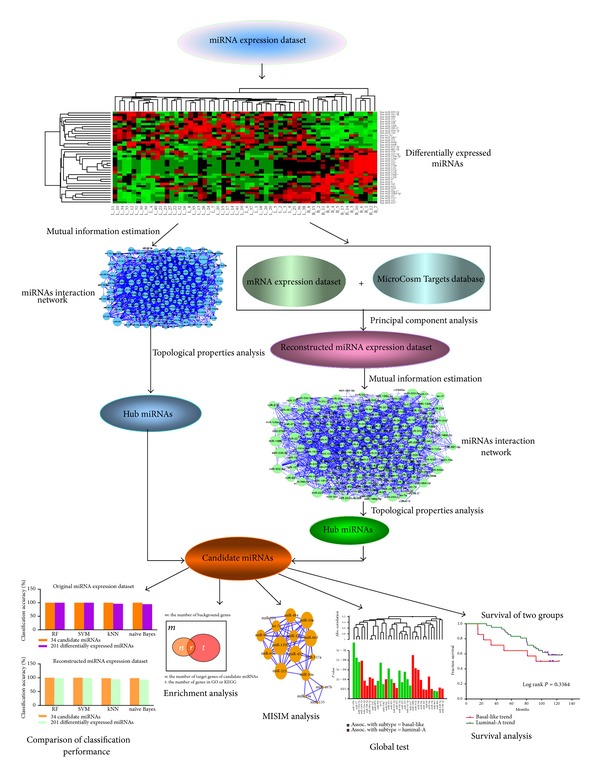
The flow chart of the method.

To explore whether candidate miRNAs extracted from two constructed miRNAs interaction networks are significantly correlated with survival, we performed Kaplan-Meier (KM) survival analysis for these candidate miRNAs. In this analysis, samples were classified using *K*-means clustering based on candidate miRNAs expression levels into two groups which were defined as luminal-A trend or basal-like trend according to the proportion of two breast cancer subtype samples. In other words, if the predicted group arising from *K*-mean cluster includes greater number of luminal-A samples than basal-like samples, this group is defined as luminal-A trend and vice versa. We used log-rank test to compare the two survival groups (luminal-A trend and basal-like trend) on the basis of the identified candidate miRNAs. The flow chart of our work was shown in Figure 1.

## 3. Results

### 3.1. Construction of miRNAs Interaction Networks

#### 3.1.1. Construction of miRNAs Interaction Network Using miRNA Expression Profiling

**Figure 2:**
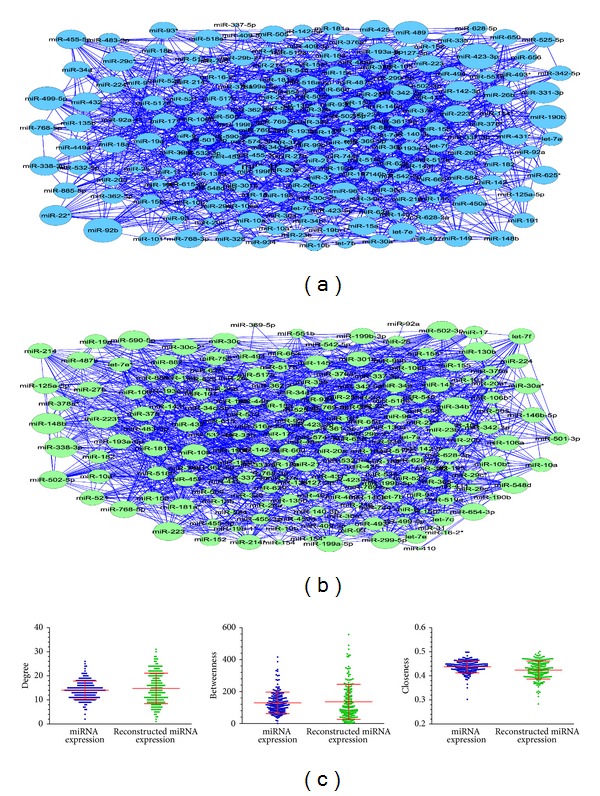
Two constructed miRNAs interaction networks. (a) miRNAs interaction network using the original miRNA expression profiling data. The larger blue circle indicates the miRNA with greater degree and vice versa. (b) miRNAs interaction network using the reconstructed miRNA expression dataset. The larger green circle indicates the miRNA with greater degree and vice versa. (c) The comparison of topological properties (average degree, average betweenness, and average closeness) between two miRNAs interaction networks.

After performing the mutual information (MI) estimation using the original miRNA expression profiling data, we obtained miRNAs interaction network in which 1,413 miRNAs interaction relationships were included. While miR-522 and miR-519a showed the strongest interaction (MI = 2.238), followed by miR-155* and miR-105 (MI = 2.176), this network was modeled as graph in which each circle node represents miRNA and each blue edge indicates the interaction between two miRNAs (see Figure 2(a)). In Figure 2(a), the larger blue circle indicates the miRNA with greater degree, whereas the smaller blue circle indicates the miRNA with smaller degree.

#### 3.1.2. Construction of miRNAs Interaction Network Using the Reconstructed miRNA Expression Dataset

For each of 201 differentially expressed miRNAs, we extracted the first principal component of all its targets. Generally, when the first principal component by itself explains less than 40% of the variance, more components should be needed (http://www.mathworks.com/help/stats/feature-transformation.html). In our analysis, the contributions of the first principal component were all more than 40%, and the minimum contribution of the first principal component was 44.7%. Therefore, for each miRNA, we used the first principal component of all its targets to represent its expression. For the reconstructed miRNA expression dataset arising from PCA, we still adopted the same mutual information estimation method to construct miRNAs interaction network. As a result, 1,466 miRNA interaction relationships were included in this network. While miR-29c* and miR-9 showed the strongest interaction (MI = 2.760), followed by miR-145* and miR-199a-5p (MI = 1.859), interestingly, miR-9 and miR-199a-5p were all potential breast cancer subtype related miRNAs supported by recent literature and clinical experiences [33, 34]. This network was also modeled as a graph in which each circle node represents miRNA and each blue edge indicates the interaction between two miRNAs (see Figure 2(b)). In Figure 2(b), the larger green circle indicates the miRNA with greater degree, whereas the smaller green circle indicates the miRNA with smaller degree. 

### 3.2. Identification of Breast Cancer Subtype Related miRNAs from Two Constructed miRNAs Interaction Networks

**Figure 3:**
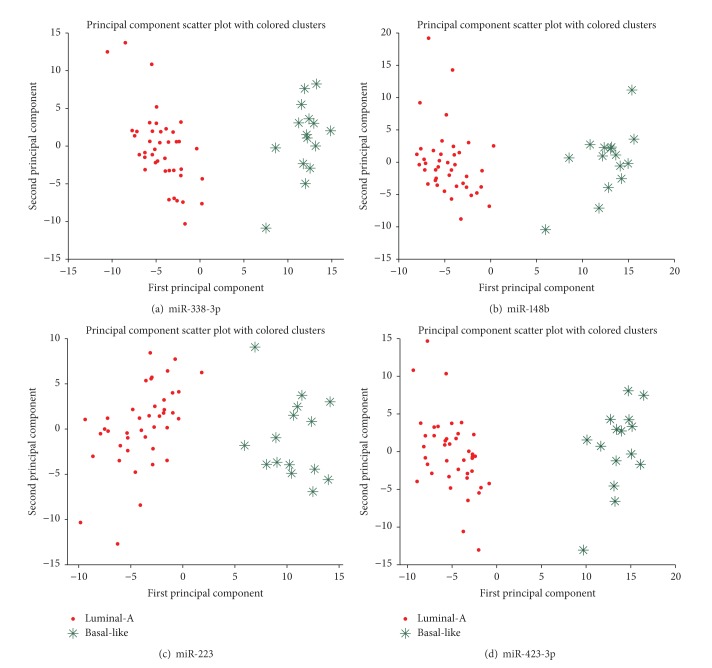
The principal components scatter plots for (a) miR-338-3p, (b) miR-148b, (c) miR-223, and (d) miR-423-3p. The red circles indicate the luminal-A samples while the green snowflakes indicate the basal-like samples.

Here, we compared the network topological properties of these two constructed miRNAs interaction networks (see Figure 2(c) and Table 1). From Figure 2(c) and Table 1, we found that the topological properties of these two networks are very similar, such as the network density (0.070 and 0.074, resp.), the network centralization (0.060 and 0.083, resp.), the average degree (14.06 and 14.81, resp.), the average betweenness (129.39 and 135.90, resp.), and the average closeness (0.437 and 0.424, resp.). Now we focused our attention on those hubs. We assumed that the degree of nodes followed a Poisson distribution in a random network [35]; we calculated the probability of *P* (degree ≥ *t*) under the null hypothesis that nodes in the network were connected randomly. The results showed that a node with degree ≥20 in a random network is a rare event (*P* < 0.05) under the null hypothesis. In order to get more information, we relax the degree threshold to 15. This assumption is consistent with some previous studies in which a protein node with degrees ≥15 in a disease related network is considered as a hub protein [36, 37]. Therefore, in this analysis, we considered those miRNAs with degree ≥15 as hubs. By comparing these two networks, 34 common hubs shared by them were identified (see Table 2). We found these hub miRNAs were all breast cancer subtype related miRNAs, while 15 miRNAs were basal-like trend and 19 miRNAs were luminal-A trend. We calculated the average degree, the average betweenness, and the average closeness of these hubs across these two networks. We found hubs with higher degree also show the higher betweenness and higher closeness. In other words, a miRNA with higher betweenness and higher closeness means that it is on higher number of shortest paths between miRNAs, and this miRNA is important [25]. Note that the first two principal components of the top ranked miRNAs, such as miR-148b, miR-223, and miR-423-3p, also classified the samples very well (see Figure 3). Some lines of literature evidence can support these results. For example, miR-148b showing the outstanding topological properties (average degree = 24, average betweenness = 356.126, and average closeness = 0.4755) was approved a potential breast cancer marker. Cuk et al. found miR-148b was significantly upregulated in the plasma of breast cancer patients [38]. For another example, miR-223 (average degree = 22.5, average betweenness = 225.153, and average closeness = 0.4645), a miRNA specific for IL-4-activated macrophages, was detected within the exosomes released by macrophages and was significantly elevated in the cocultivated SKBR3 and MDA-MB-231 cells [39]. The invasiveness of the cocultivated breast cancer cells decreased when the IL-4-activated macrophages were treated with a miR-223 antisense oligonucleotide (ASO) that would inhibit miR-223 expression. In addition, some other miRNAs showing the outstanding topological properties were also approved to be potential breast cancer or breast cancer subtype related miRNAs, such as miR-423-3p (average degree = 21.0, average betweenness = 259.025, and average closeness = 0.4735), which was found to be associated with the disease subtype and the survival of breast cancer patients [40].

**Figure 4:**
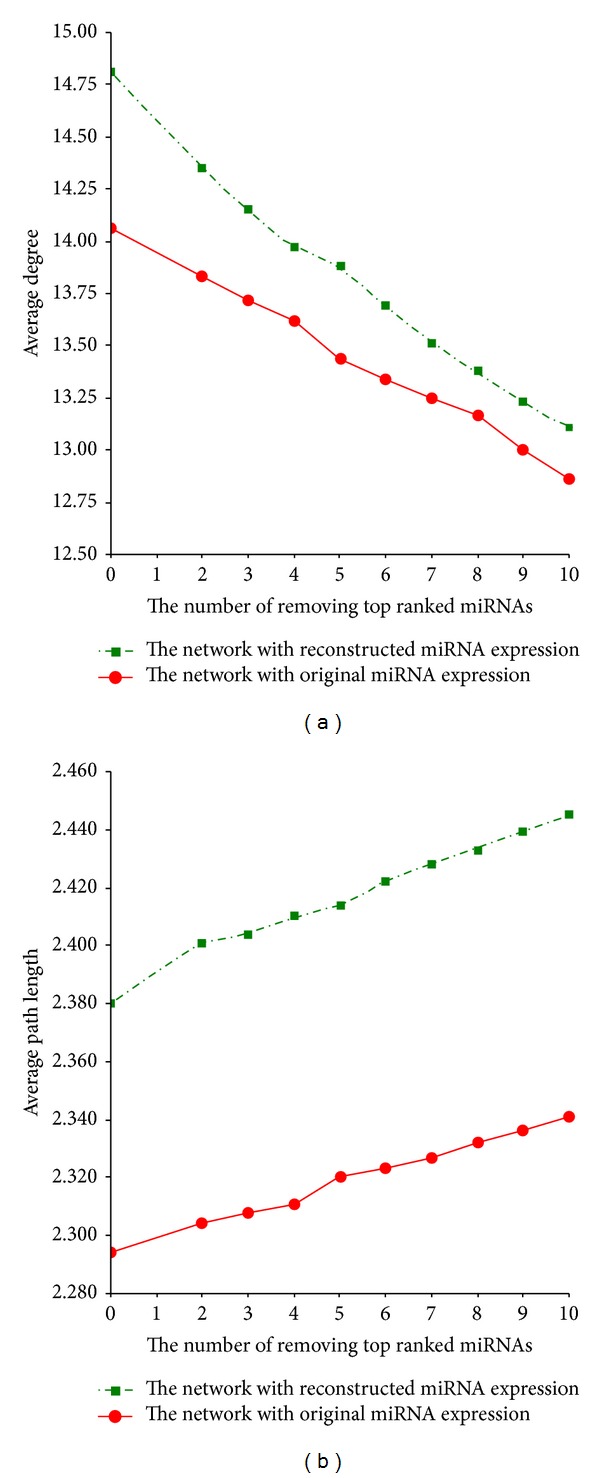
The change of the network topological properties as the top 10 ranked miRNAs in the degree are removed sequentially from two individual miRNAs interaction networks. (a) The change of the network average degree. (b) The change of the network average path length.

In order to explore whether removing important miRNAs can lead to the special network properties change, we removed the top 10 ranked miRNAs in the degree sequentially from two individual networks and observed the change in the networks topological properties. After removing the top 10 ranked miRNAs with higher degree, the average degree of the network based on the original miRNA expression dataset and the network based on the reconstructed miRNA expression dataset decreased from 14.06 to 12.86 and from 14.81 to 13.11, respectively, whereas the average path length increased from 2.294 to 2.341 and from 2.380 to 2.445, respectively. In other words, after removing the top 10 ranked miRNAs sequentially, we cannot find the obvious change of the network topological properties, and the average path length increased smoothly as the average degree decreased smoothly for these two miRNAs interaction networks (see Figure 4). Therefore, whether the important miRNA or miRNA clusters can predominate in the network topological properties needs to be validated. Finally, the identified 34 common hub miRNAs showing the outstanding topological properties will be as candidate miRNAs coming into our further analysis.

### 3.3. Comparison with Other Methods

#### 3.3.1. Random Test

**Figure 5:**
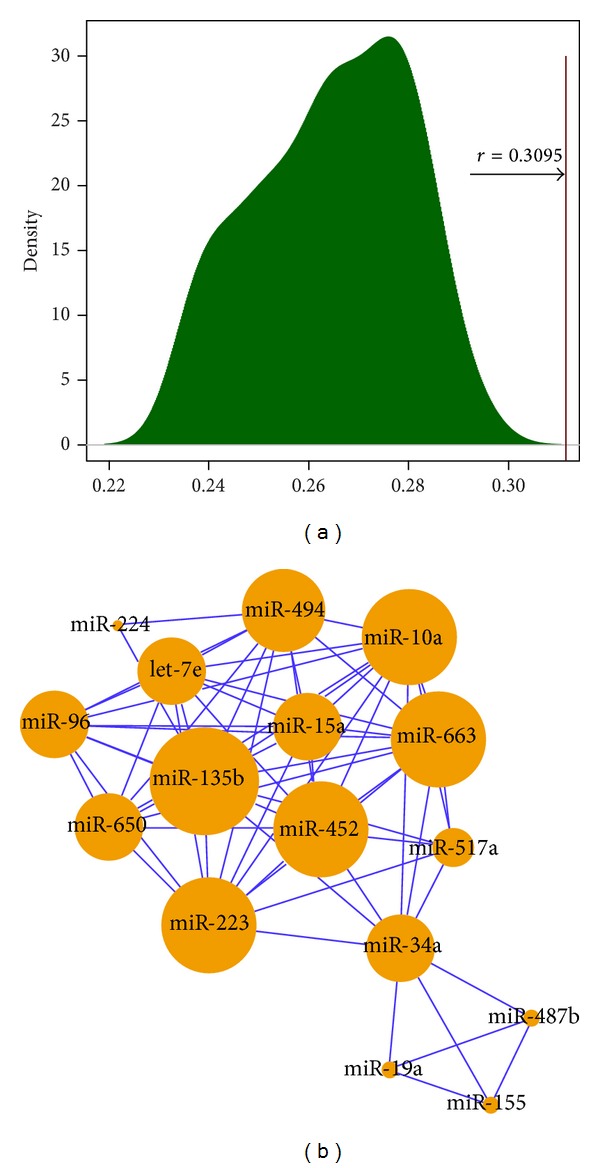
(a) Random test. Randomly chose 34 breast cancer related miRNAs 1,000 times and calculated their average correlation coefficients in each random condition. The average correlation coefficients in each random condition are lower than the average of correlations of 34 candidate hub miRNAs (*r* = 0.3095). (b) miRNA cluster acquired with MISIM tool. The larger orange circle indicates the miRNA with greater degree, whereas the smaller orange circle indicates the miRNA with smaller degree.

To validate whether the identified 34 hub miRNAs have higher similarity than general breast cancer related miRNAs, we download 86 breast cancer related miRNAs with the key word of “breast cancer” by searching the miR2Disease database (http://www.mir2disease.org/) which is a manually curated database that aims to provide a comprehensive record of miRNA deregulation involved in various human diseases [41, 42]. When these miRNAs are mapped into the miRNA expression dataset used in this paper, 57 miRNAs with the corresponding expression values were obtained. From these 57 miRNAs, we randomly selected 34 miRNAs 1,000 times and calculated their average correlation coefficients in each random condition. We found that none of the average correlation coefficients in each random condition is higher than the average of correlations of 34 candidate hub miRNAs (*r* = 0.3095), and the maximum average correlation coefficient is 0.2732 in random conditions (see Figure 5(a)). Therefore, this result supports the assumption that candidate hub miRNAs might have potential similar function.

#### 3.3.2. Comparison with MISIM Tool

To further validate whether the identified miRNAs have the similar function, we used a miRNA similarity (MISIM) tool [9] to measure the functional similarity of 34 shared hub miRNAs based on human miRNA-disease association data and the structures of the corresponding disease relationships. We used the recommended MISIM threshold of 0.7 to determine whether two miRNAs have a link. In other words, those miRNA pairs with MISIM coefficient greater than or equal to 0.7 will be selected. The results of MISIM analysis showed that miR-223, miR-452, let-7e, miR-10a, miR-663, and miR-15a had a similar function (see Figure 5(b)). Indeed, a few of the newly published literature have approved some of these miRNAs are associated with breast cancer subtype, such as miR-223. These results suggest miRNA clusters identified by our method might have potential functional congregation related to breast cancer subtype.

#### 3.3.3. Comparison with Our Previous Results

In addition, we also compared these candidate miRNAs with our previously identified miRNAs which were obtained from the constructed luminal-A trend and basal-like trend miRNA-miRNA network based on the defined correlation coefficient ratio (CCR) [19]. We found that four identified common miRNAs (miR-199a-5p, let-7e, miR-342-3p, and miR-125a-5p) were all associated with breast cancer subtype. Also, in the acquired clusters (modules) of highly correlated miRNAs using the weighted correlation network analysis (WGCNA) method [19, 43], it is interesting to find that the candidate hub miRNAs showed the similar expression, such as let-7e and miR-125a-5p; miR-182 and miR-96; miR-17* and miR-19a; and miR-142-3p, miR-155, miR-146b-5p, and miR-223.

### 3.4. Comparison of Subtype Classification Performance

As we expected, for the original miRNA expression profiling data, the classification accuracy of miRNAs group with 34 common miRNAs shared by two miRNAs interaction networks is up to 100% using four classifiers. The classification accuracy of miRNAs group with 201 differentially expressed miRNAs is 100%, 100%, 96.4%, and 94.6% for RF, SVM, *k*NN, and naïve Bayes classifiers, respectively. For the reconstructed miRNAs expression data arising from PCA, the classification accuracy of miRNAs group with 34 common miRNAs shared by two miRNAs interaction networks is 100.0%, 100.0%, 98.2%, and 98.2% for RF, SVM, *k*NN, and naïve Bayes classifiers, respectively. The classification accuracy of miRNAs group with 201 differentially expressed miRNAs is 98.2%, 98.2%, 94.6%, and 92.8% for RF, SVM, *k*NN, and naïve Bayes classifiers, respectively. It is well known that RF and SVM classifiers have a higher classification performance than *k*NN and naïve Bayes classifiers [31]. In other words, two classifiers with slightly lower performance all showed that the miRNAs group with 34 common miRNAs shared by two miRNAs interaction networks was more powerful than the other group when used as predictor variables to classify samples. This result supports our hypothesis and can indicate that some hub miRNAs showing the outstanding topological properties in the disease network might contribute to disease or disease subtype or serve as predictive biomarkers and effective targets for therapeutic intervention.

### 3.5. GO and KEGG Functional Enrichment Analysis

In this analysis, for each of identified candidate miRNAs, we used DAVID (http://david.abcc.ncifcrf.gov/) to perform GO and KEGG functional enrichment analysis for its targets, and a GO term (or a KEGG pathway) with a *P*  value of 0.01 was considered to be significant. We did not perform the multiple test correction to avoid a loss of true-positive results. The KEGG enrichment analysis results showed that the targets of the identified candidate miRNAs were significantly enriched on the functions related to amino acid metabolism, such as pyrimidine metabolism and histidine metabolism. This is in agreement with the previous findings that miRNAs selectively regulate certain metabolic processes such as amino acid biosynthesis, so that they can selectively control certain metabolite production [44]. GO enrichment analysis results showed that the function of genes targeted by some shared hub miRNAs, such as miR-15a and miR-199a-5p, focused on protein kinase activity. 

### 3.6. A Global Test for 34 Common Hub miRNAs Shared by Two miRNAs Interaction Networks

**Figure 6:**
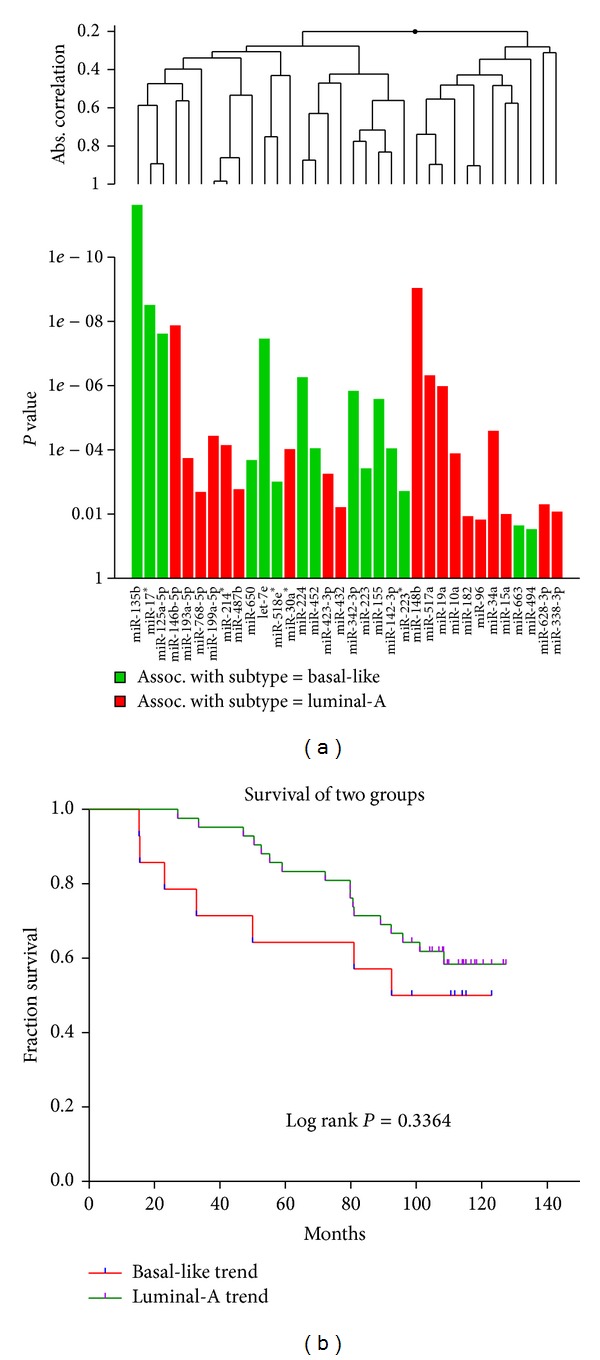
(a) Global test for 34 candidate hub miRNAs shared by two constructed miRNAs interaction networks. This graph is based on the decomposition of the test statistic into the contributions made by each of miRNAs in the alternative hypothesis. The graph illustrated the *P* values of the tests of individual component miRNAs of the alternative. The plotted miRNAs are ordered in a hierarchical clustering graph. The clustering method is average linkage. (b) Survival analysis for 34 candidate hub miRNAs shared by two miRNAs interaction networks. Samples were classified using *K*-means clustering based on the expression levels of 34 candidate miRNAs into two groups which were defined as luminal-A trend or basal-like trend according to the proportion of two breast cancer subtype samples.

To explore whether the identified 34 candidate hub miRNAs are associated with breast cancer subtype, we used Goeman's global test here to determine its significance. The results showed that this candidate miRNAs set is strongly associated with the breast cancer subtype (*P* = 1.05*E* − 23) (see Figure 6(a)). When we selected the top 10 hub miRNAs to perform the same analysis, the strong association still existed (*P* = 1.96*E* − 11). From Figure 5(a), we can see that miR-135b displays a strong association with breast cancer subtype (*P* = 2.35*E* − 12, FDR = 2.30*E* − 10) and shows the obvious basal-like trend (the average expression in basal-like samples is 2.231 times as that in luminal-A samples). Recent evidence has approved miR-135b is upregulated in basal-like tumor subtypes [17]. Moreover, an interesting observation was that miR-34a showed an association with luminal-A subtype (*P* = 2.55*E* − 05, FDR = 0.00024). Recent studies found the tumors with high expression of miR-34a represented aggressive breast cancers but the tumors with lower expression suffered from significantly increased tumor recurrence [45]. Thus, miR-34a presents a novel and peculiar finding which needs to be explored in future studies [46].

### 3.7. Survival Analysis for 34 Candidate Hub miRNAs Shared by Two miRNAs Interaction Networks

To explore whether the identified 34 candidate miRNAs are significantly correlated with survival, we performed Kaplan-Meier (KM) survival analysis for these candidate miRNAs. The analysis found that the two groups (luminal-A trend and basal-like trend) arising from *K*-mean cluster did not display the obvious different survival rate (log rank *P* = 0.3364; see Figure 6(b)). This result agrees with Enerly et al.'s study in which they did not find any significant association of miRNAs to survival in the entire cohort except miR-150 which was found to be predictive of better prognosis within the corresponding set of patients in part of the cohort [17]. Maybe an increased sample size can change this case.

## 4. Discussion

As we know, cancer is the result of a complex multistep process that involves the accumulation of sequential alterations of several genes, including those encoding microRNAs (miRNAs). A large body of evidence has implicated that aberrant miRNA expression patterns exist in most of human malignancies. A single miRNA might have many targets that are involved in different oncogenic pathways, and a small group of miRNAs are consistently deregulated in a wide variety of hematological malignancies and solid tumors; developing strategies to silence or reexpress these miRNAs will likely affect several groups of patients [47]. These findings suggest that miRNA profiling has diagnostic and perhaps prognostic potential [48, 49]. 

In this paper, we used a novel mutual information estimation method to construct two miRNAs interaction networks based on miRNA-mRNA dual expression profiling data and identified the common hub miRNAs shared by these two networks, some of which were approved to be breast cancer subtype related miRNAs. A key difference between our method and other network-based methods is that we constructed two miRNAs interaction networks utilizing miRNA-mRNA dual expression profiling information arising from the same samples and identified the common miRNAs showing the outstanding topological properties in both of the two networks. Specifically, we know that the detection of dependencies between biology random variables is highly useful in feature selection, such as biomarker identification. However, many dependencies between biomarkers are not linearly correlated, and the classical correlation analysis cannot be used for discovering nonlinear dependencies with no correlation. Therefore, as a powerful method, mutual information plays an important role in information theory which allows us to identify general nonlinear dependencies between biomarkers [50]. Our analysis integrated miRNA-mRNA target relationships, principal component analysis, and mutual information estimation, which will enhance the power for identifying disease-related or disease subtype related miRNAs. This study provides a new analyzing method from system biology level and helps to understand the relationship between miRNA and mRNA in primary breast cancer subtype.

A noteworthy observation is that not all differentially expressed miRNAs can be identified as breast cancer subtype related although miRNA expression alone is sufficient to distinguish luminal-A from basal-like samples [17]. Therefore, the joint analysis of miRNA and mRNA utilizing their dual expression profiling information will make the findings more accurate. Moreover, it is interesting to obtain some additional information from this analysis. For example, among the 34 common hub miRNAs shared by two miRNAs interaction networks we found that the let-7 family (let-7e) and miR-342 family (miR-342-3p) were included. Indeed, these miRNAs displayed a more significant differential expression between TP53 mutational statuses than between estrogen receptor (ER) statuses [17] and have previously been linked to tumorigenesis [51, 52].

We should point out the limitations of this analysis. In the present study, we only analyzed the predicted direct miRNA-target regulation owing to the computational complexity of miRNA-mRNA relationships. In the practice, many predicted algorithms focus on a similar feature set for their prediction under the hypothesis that all miRNA target sites are evolutionary conserved. Unfortunately, not all miRNA target sites are conserved or adhere to canonical seed complementarity [53]. Therefore, using the predicted miRNA-target regulation may have the potential impact on the final results. Specifically, the lack of miRNA-mRNA dual expression profiling datasets of breast cancer subtype and the relative small sample size cause the limitations in the data analysis, and the results need to be approved in the future studies when more miRNA-mRNA dual expression profiling datasets of breast cancer subtype are available.

## 5. Conclusion

In conclusion, utilizing miRNA and mRNA dual expression profiling information to perform data analysis can help reveal important findings with regard to the underlying molecular mechanisms of breast cancer subtype and also help to identify candidate breast cancer subtype related miRNAs using the distinct network properties.

## Figures and Tables

**Table 1:** The comparison of network topological properties between two miRNAs interaction networks.

Network topological properties	Using the original miRNA expression dataset	Using the reconstructed miRNA expression dataset arising from PCA
Network edge	1,413	1,466
Avg. degree	14.06	14.81
Avg. betweenness	129.39	135.90
Avg. clustering coefficient	0.072	0.177
Avg. closeness	0.437	0.424
Network density	0.070	0.074
Network heterogeneity	0.268	0.439
Network centralization	0.060	0.083
Characteristic path length	2.294	2.380
Network diameter	4	5
Network radius	3	3

Avg: average.

**Table 2:** 34 common hub miRNAs shared by two miRNAs interaction networks.

miRNA	Avg. degree	Avg. betweenness	Avg. closeness
miR-338-3p	25.0	357.936	0.4865
miR-148b	24.0	356.126	0.4755
miR-223	22.5	225.153	0.4645
miR-223*	22.5	225.015	0.4645
miR-423-3p	21.0	259.025	0.4735
miR-768-5p	21.0	248.056	0.4645
miR-125a-5p	20.5	218.049	0.4670
miR-432	20.5	228.476	0.4590
miR-193a-5p	20.0	182.244	0.4640
miR-487b	20.0	314.999	0.4640
let-7e	19.5	229.821	0.4575
miR-142-3p	19.5	175.504	0.4600
miR-199a-5p	19.5	247.286	0.4695
miR-19a	19.5	137.946	0.4645
miR-224	19.5	217.737	0.4725
miR-30a*	19.5	190.177	0.4590
miR-452	19.5	281.325	0.4710
miR-146b-5p	19.0	241.451	0.4600
miR-34a	19.0	133.652	0.4540
miR-10a	18.5	161.993	0.4495
miR-135b	18.5	168.629	0.4600
miR-182	18.5	135.153	0.4525
miR-214*	18.5	177.383	0.4460
miR-517a	18.5	143.098	0.4600
miR-15a	18.0	211.751	0.4530
miR-628-3p	18.0	203.516	0.4475
miR-96	18.0	205.601	0.4525
miR-17*	17.5	128.604	0.4505
miR-342-3p	17.5	185.313	0.4470
miR-518e*	17.5	150.067	0.4515
miR-155	16.0	121.071	0.4420
miR-494	16.0	220.821	0.4430
miR-650	16.0	169.994	0.4490
miR-663	15.0	98.169	0.4350
